# Evaluation of immunomodulatory activities of methanolic extract of khat (*Catha edulis,* Forsk) and cathinone in Swiss albino mice

**DOI:** 10.1186/s12865-015-0072-5

**Published:** 2015-02-22

**Authors:** Tsige Ketema, Moti Yohannes, Esayas Alemayehu, Argaw Ambelu

**Affiliations:** Department of Environmental Health Sciences and Technology, Jimma University, College of Public Health and Medical Sciences, Jimma, Ethiopia; Jimma University Institute of Technology, School of Civil and Environmental Engineering, Jimma, Ethiopia; Department of Biology, Jimma University, College of Natural Sciences, Jimma, Ethiopia; Department of Microbiology and Veterinary Public Health, Jimma University, College of Agriculture and Veterinary Medicine, Jimma, Ethiopia

**Keywords:** CD4^+^, CD8^+^, Humeral immunity, Lymphocyte, Phagocytic index, WBC

## Abstract

**Background:**

The objective of this study was to explore the immunomodulatory effect of khat and its active component, cathinone, using mice.

**Materials and methods:**

Female Swiss albino mice aged 7–8 weeks weighing 25-30 g were used in the study. Mice were randomized into eight groups of 6 each and oral treatment of khat crude extract and cathinone were given daily for four weeks. Physical, hematological, biochemical, and immunological parameters were measured. Immunological studies included humeral immunity (IgG and IgM), cellular immune response (delay type hypersensitivity), phagocytic activities of reticuloendothelial system, and determination of T-lymphocyte population: CD3^+^, CD4^+^, CD8^+^ count and CD4^+^/CD8^+^ status.

**Results:**

Findings of this study showed that, khat and its major metabolite, cathinone, can positively affect immune system in dose dependent manner. When doses of crude khat extract and cathinone increase, the induction of humeral (IgG and IgM) and cellular immune responses were up-regulated significantly (P < 0.05), while at higher dose of khat (200 mg/kg) cellular immune response was suppressed. In support of this, as doses of the two test substances increased, the count of T helper cells (CD4^+^) was significantly increased (P < 0.05), while higher dose significantly reduced whole white blood cell (WBC), CD8^+^, and CD3^+^ counts.

**Conclusion:**

At relatively lower dose (50-100 mg/kg), crude khat extract has immune stimulating property, although higher dose (200 mg/kg) leads to suppression of cellular immune response. Cathinone also share all immune modulating property of its parent compound, khat, but with intense strength. Thus, it calls for further detailed investigation of khat for practical application of the same in human medication.

**Electronic supplementary material:**

The online version of this article (doi:10.1186/s12865-015-0072-5) contains supplementary material, which is available to authorized users.

## Background

Khat (*Catha edulis*, Forsk) is an evergreen shrub, primarily cultivated for its use as a natural stimulant. It is chewed by millions of people in Yemen, Somalia, Ethiopia, Djibouti and Kenya [1]. Besides its use as a social and recreational stimulant, traditionally people use processed leaves and roots of khat for treatment of various ailments including influenza, cough, asthma, malaria, gonorrhea, vomiting and headache [1,2].

Stimulating effect of khat is mainly due to its content of the alkaloid, cathinone, and to a lesser extent the diastereomers, cathine and norephedrine [3]. Cathinone is an intermediate metabolite in the biosynthesis of cathine and is found mainly in young fresh leaves of the khat plant [3]. It is relatively unstable and decomposes within few days of picking or if the leaf is dried it changes into norpseudoephedrine and norephedrine [4].

About 100-200 g of fresh leaves or shoots of khat are chewed per session and juice of the khat is swallowed while the plant residue is retained in mouth [5]. Khat induces a mild euphoric state giving the chewer a feeling of being more focused, energetic and communicative [1,4]. Thus, daily laborers, farmers and students use khat during work in order to increase their alertness and reduce physical fatigue [1,4].

On the other hand, khat is known to increase susceptibility to cognitive impairment, cardiovascular disorders, stomach ulcer, increase adrenocorticotrophic hormone levels, urine retention, gastro-intestinal tract constipation and hemorrhage due to tannin and norpesudoephedrine content of the plant [6-8]. Cathinone also increases heart rate, arterial blood pressure and respiratory rate transiently. It also improves cerebral blood flow, mental alertness and makes individuals energetic [1,3]. Cathinone is structurally related to the synthetic drug, amphetamine, and has been shown to have a similar pharmacological profile [9]. It induces central nervous system and peripheral effects like euphoria, hyperactivity, restlessness, increase alertness, joyful state, reduce physical fatigue on users, cause mouth dryness, mydriasis, anorexia, hyperthermia, hypertension and tachycardia [3,4]. Despite all these reports on psychological and physiological effects of khat and cathinone, its immune modulatory roles were not investigated in depth. Thus, this study was designed to assess effect of khat and cathinone on immune system, using Swiss albino mice as animal model.

## Methods

### Experimental animal

Female Swiss albino mice, aged 7–8 weeks weighing 25-30 g, kindly obtained from Ethiopian public health institute (EPHI), Addis Ababa, were used in this experiment. The mice were housed in transparent plastic cage having SS sipper 250 ml water bottle. Wood shaving was used as bedding and it was replaced every morning having cleaned and disinfected the cage with 70% alcohol. Animals were kept under unlimited access to commercial pellet food and fresh tap water *ad libitum.* The experimental room has 12/12 hrs light/dark cycles, 55% ± 5.7 humidity, and mean temperature of 21°C.

### Collection and extraction khat

Fresh khat leaves were purchased from local market. Its shoot and leave were carefully picked and chopped into small pieces (~0.5 cm^2^) using grinding machine and mixed in methanol (analytical grade). For 100 g of the plant, 300 ml of methanol was used. First phase extraction was carried out by spinning the mixture for 15 minutes in Water Bath Shaker (RSB - 12) at 120 rpm at room temperature under dark condition. The mixture was filtered using whatmann filter paper (11 μm pore size) and the residue was re-shaked overnight under the same condition of the former filtrate. Then, the filtrate was concentrated to near dryness using Rota vapor (Heidolph Labo Rota, 4002) at 120 rpm and temperature of 40°C for 1-2 hrs. The residue was measured and dissolved in 20% Tween 80 in physiological saline solution.

Freshness of the khat extract was checked on thin layer chromatography (TLC) following procedure developed by Lee [10]. Briefly, the plant extract was spotted directly onto a pre-coated *silica gel 60* plate. Cathinone oxalate and cathine oxalate drug standards dissolved in methanol was used as references. The plate was developed in solvent composition of ethyl acetate: methanol: aqueous ammonia (85:10:5), and viewed under an ultraviolet lamp (254 nm). The spots were visualized using 0.5% ninhydrin solution, and then the plate was heated. Cathine appeared purple, while cathinone was a burnt orange moving spot. The retardation factor (R_f_) values obtained for cathinone and cathine were 0.43 and 0.21, respectively.

Cathinone isolated from khat (*Catha edulis*, Forsk) was obtained from African Laboratory for Natural Products, Department of Chemistry, Addis Ababa University, Ethiopia. Purity of the cathinone was checked prior to treatment by running proton and carbon NMR spectra (Additional file 1).

### Khat and cathinone acute toxicity test

Eighteen female Swiss albino mice were randomly divided into 3 groups of 6 mice each. After being fasted for 2 h [11,12], the first and the second groups were treated orally with 100 and 200 mg/kg body weight (BW) of crude khat extract, respectively, while the third group with 5 mg/kg BW cathinone. Treatment was given daily for two weeks and mice were monitored for any changes including loss of appetite, hair erection, lacrimation, tremors, convulsions, diarrhoea, salivation, mortality and other signs of observable toxicity [12].

### Mice grouping and treatment

After a week acclimatization period in laboratory, mice were grouped into eight categories, each containing six mice. Groups I-IV received dose of 0.625, 1.25, 2.5 and 5 mg/Kg cathinone following doses used by Qureshi et al. [13], whereas groups V-VII received dose of 50, 100 and 200 mg/kg khat following a dosage used by Al-meshal et al. [14] for khat toxicity test. Mice in category VIII were treated with 20% Tween 80 in physiological saline solution as a control group. Accordingly, all groups of mice received their respective treatments daily using oral gavage for 4 weeks. The control group also received 0.5 ml of 20% Tween 80 in saline solution through oral route for similar duration and frequency.

### Effect on immune organs

Body weight of each mouse in the experimental (I-VII) and control group were taken every week. On the next day of the last dose, each mouse was terminally anesthetized and relative organ weight (thymus, and spleen) was measured. Then, relative organ weight was calculated by dividing weight of each organ to body weight of the mice and multiplied by 100. Further effects on liver and kidney weight were assessed.

### Hematological and biochemical tests

From lethally anesthetized mice, experimental and control, blood sample was collected on the next day of the last dose using cardiac puncture into a sterile EDTA coated tube. Aliquot (~10 μL) of the blood sample was used for quantification of total WBCs, lymphocytes, monocytes, RBCs, Haemoglobine (Hb), Hematocrit (HCT), platelets, mean corpuscular volume (MCV), mean corpuscular hemoglobin concentration (MCHC), and mean corpuscular hemoglobin (MCH) using CBC machine (Automated CBC Analyzer: Sysmex KX-21). The blood sample was also used to run liver and kidney function test. Accordingly, serum was separated by centrifuging blood at 3,000 rpm for 10 minutes. The supernatant was transferred into new eppendorf tube and immediately analyzed for liver enzymes level such as serum glutamic oxaloacetate transaminase (sGOT) and serum glutamic pyruvic transaminase (sGPT) in addition to the determination of albumin levels. Moreover, kidney function tests such as quantification of creatinine and urea were made using the serum collected from liver biomarkers test using Axsym MEIA 3rd Generation immunochemical automated analyzer, Abbott diagnostics.

### Activities of khat and cathinone on immune system

#### Humeral immunity

Effects of the two test substances on antibody production were determined by hemagglutination titer (HT) test [15]. For this assay, the previous mice grouping pattern was followed although the activities differ. All mice were immunized with 0.1 ml of 1 × 10^9^ sheep red blood cells (SRBCs, Sigma Aldrich) in PBS (pH = 7.2) intra-peritonially (ip) on the 28^th^ day of treatment administration. This day was designated as day 0. Then, treatment of sensitized mice with cathinone or crude khat extract was extended to day 4. Anti-sheep RBC haemagglutinin titer was determined on day 4 post SRBC immunization following procedure used by Yi *et al*. [15]. Blood sample was collected from anaesthetized mice. The sera were collected by centrifugation of whole blood at 3,000 rpm for 10 min and then inactivated at 56°C for 30 min. The antibody titer was determined by a two-fold serial dilution of one volume (100 μL) of serum and one volume (100 μL) of 0.1% SRBCs in bovine serum albumin (BSA). Then, the tubes were incubated for 30 minutes at 37°C. The total and 2-mercaptoethanol resistant serum agglutination titers were defined by an active haemagglutination test according to the procedure stated by Adler [16]. This author described that the titer of 2-mercaptoethanol-resistant antibody was roughly equivalent to that due to IgG in the serum, and thus the greater titer obtained without 2-mercaptoethanol was due to IgM. Hemagglutination titer was converted into mean log_2_ value for the purpose of analysis as used by Somarathna *et al*., [17].

### Cellular immunity

Treatment and primary immunization of mice with SRBCs for determination of delay type hypersensitivity reaction (DTH) was conducted following the procedure used for assessment of humeral immunity. But, in DTH reaction, secondary immunization was made through subcutaneous administration of SRBCs in the left hind footpad while the right hind paw received physiological saline solution. After 48 hours of post injection, difference between left and right paw thickness/swelling was measured using digital caliper (Mitutoyo Vernier Caliper, Japan). Dexamethason (0.2 mg/kg daily) was given for positive control mice.

#### Carbon clearance assay

Phagocytic activities of reticulo-endothelial systems were assessed by carbon clearance assay following procedure used by Hudson and Hey [18]. Mice grouping pattern was the same to the one described above. Here, on the next day of final dose, all mice received 0.1 ml Indian ink (1:50) maintained at 37°C, intravenously through tail. Blood samples were collected from the animals in small size tube coated with 5μL EDTA (0.6 mg EDTA/10 ml of DH_2_O) at 0 and 15 minutes of the treatment. From each mouse, 50 μL of blood sample was pipetted into 4 ml of 0.1% sodium carbonate solution to lyse the RBCs. Then, absorbance of the samples was measured at 650 nm using spectrophotometer [18]. Phagocytic index (k) was calculated using the following formula:

$$ \begin{array}{l}\mathrm{k} = \left( \log\ \mathrm{e}\ \mathrm{O}\mathrm{D}1- \log\ \mathrm{e}\ \mathrm{O}\mathrm{D}2\right)\kern1.75em \\ {}\kern4em 15\  \min \end{array} $$

Where, OD1 is absorbance measured at 0 minute, and OD2 is absorbance measured at 15 minute.

##### Estimation of T-lymphoceytes subset

Some volume of the blood sample collected for the previous assay was used for quantification of T lymphocyte subsets, namely CD3^+^ (total T lymphocytes), CD4^+^ (T-helper cells), CD8^+^ (cytotoxic T-cells), and CD4/CD8 ratio using flow cytometric technology [19]. Accordingly, 100 μL of Fluoroisothiocyanate (FITC) labeled anti-mouse monoclonal antibody that react against receptors of CD3^+^, CD4^+^, and CD8^+^ were added directly to 100 μL of whole blood, which were then lysed using BD FACS lysing solution (1 in 10 ddH_2_O). Following centrifugation at 4°C for 5 min at 1500 rpm, samples were re-suspended in PBS (PH = 7.4) [20] and analyzed directly using flow cytometer.

### Data analysis

Data were checked for their completeness, correctness, and then double entered into Microsoft Office Excel (2007) sheet and then analyzed using SPSS software. Data was expressed in mean ± standard error of mean (SEM), otherwise indicated. One-way analysis of variance (ANOVA) followed by Tukey’s HSD *post-hoc* test was employed to compare effect of khat and cathinone on different variables. Values of p < 0.05 were considered statistically significant. All assays were repeated three times.

### Ethical consideration

The study was ethically approved by Ethical Review Committee of College of Public Health and Medical Sciences of Jimma University, Ethiopia. Detailed experimentation procedures in mice as described in EPHI animal handling and treatment guidelines was carefully followed.

## Results

### Acute toxicity study

The acute toxicity study showed that the tested doses of crude khat (100 and 200 mg/kg) extract and cathinone (5 mg/kg) caused no mortality within the first 24 h as well as for the following two weeks. Physical and behavioral observations of the experimental mice also indicated no visible signs of e toxicity.

#### *Effect of khat and cathinone* on body and organ weights

**Figure 1:**
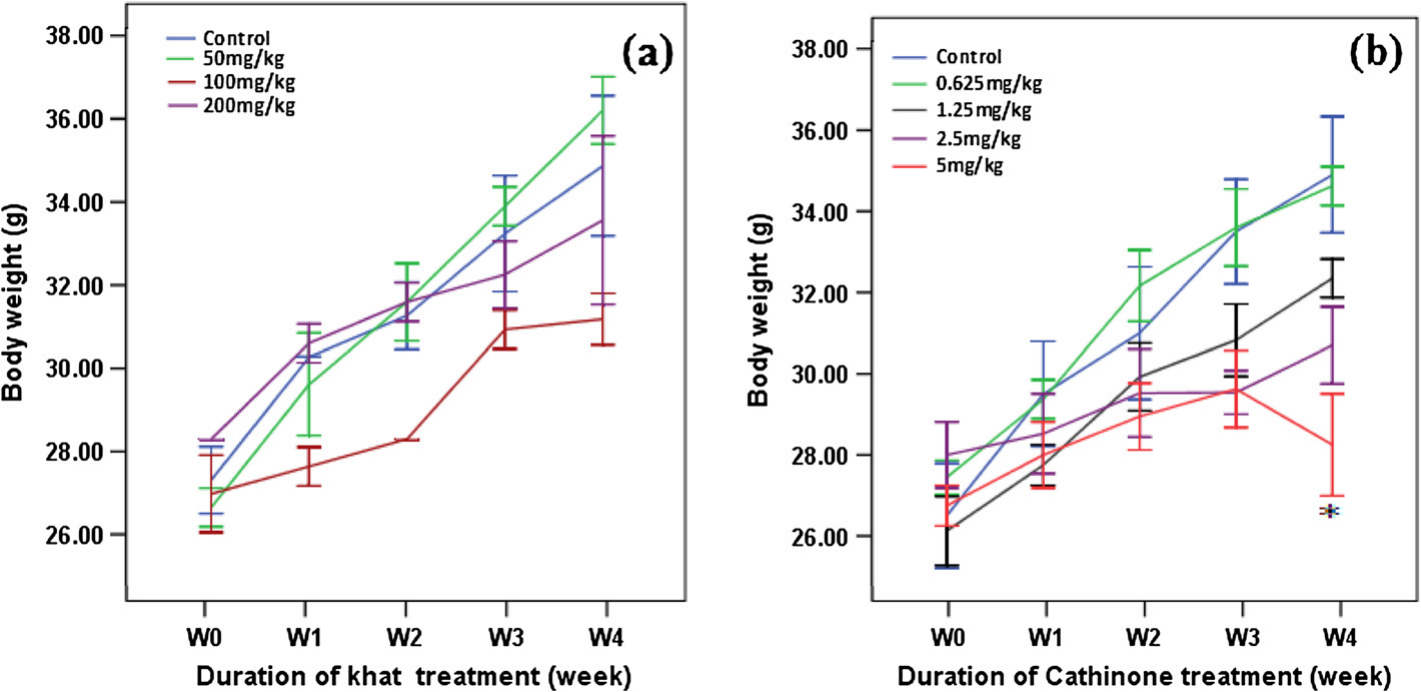
**Body weight (mean ± SEM) of Swiss albino mice (n = 6) treated with crude khat extract (a) and cathinone (b) daily for 4 weeks.** Value with asterisk is significantly different (ANOVA, Tukey’s HSD *post-hoc* test) from values of the control.

Body weight of mice treated with different doses of khat extract and cathinone didn’t show significant differences (P > 0.05) from the control mice over the four weeks follow-up period. However, in mice treated with higher dose of cathinone (5 mg/kg), significant body weight reduction was observed (P < 0.01) in the last week of the treatment (Figure 1).

**Figure 2:**
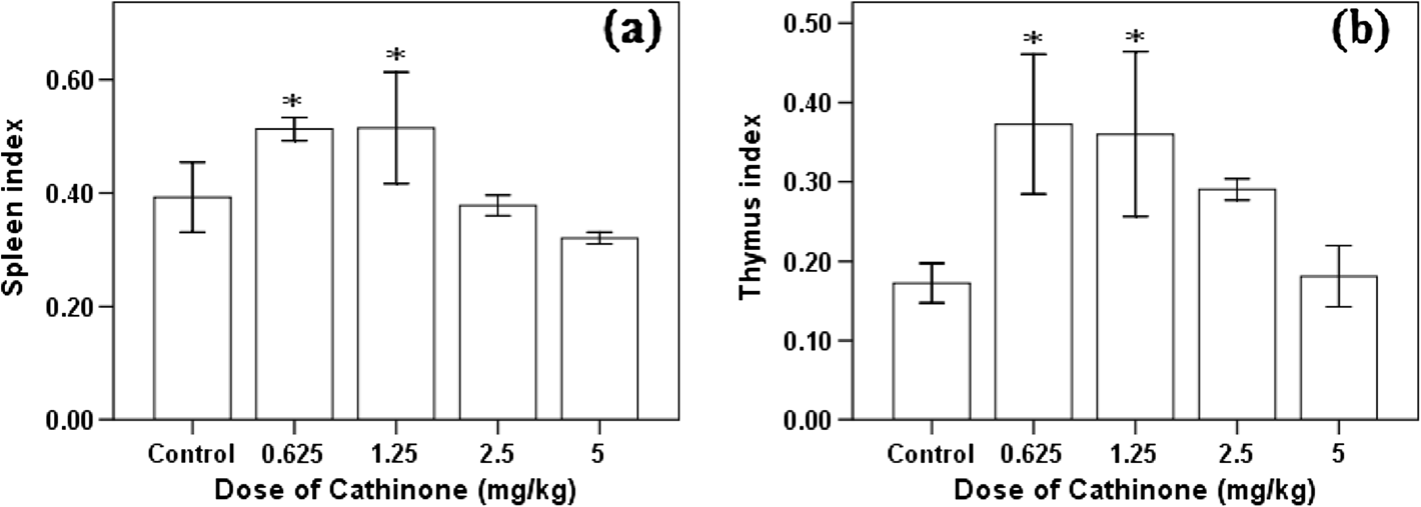
**Spleen (a) and thymus (b) index (mean ± SEM) of Swiss albino mice (n = 6) treated with cathinone daily for 4 weeks.** Values with asterisk are significantly different (ANOVA, Tukey’s HSD *post-hoc* test) from values of control.

Cathinone affected the relative organ weight of mice with dose dependent manner. As dose of cathinone increased (2.5 and 5 mg/kg), weight of organs such as spleen and thymus reduced. But significant increment (P < 0.05) of index of both organs was observed in mice that received 0.625 and 1.25 mg/kg of cathinone (Figure 2).

**Figure 3:**
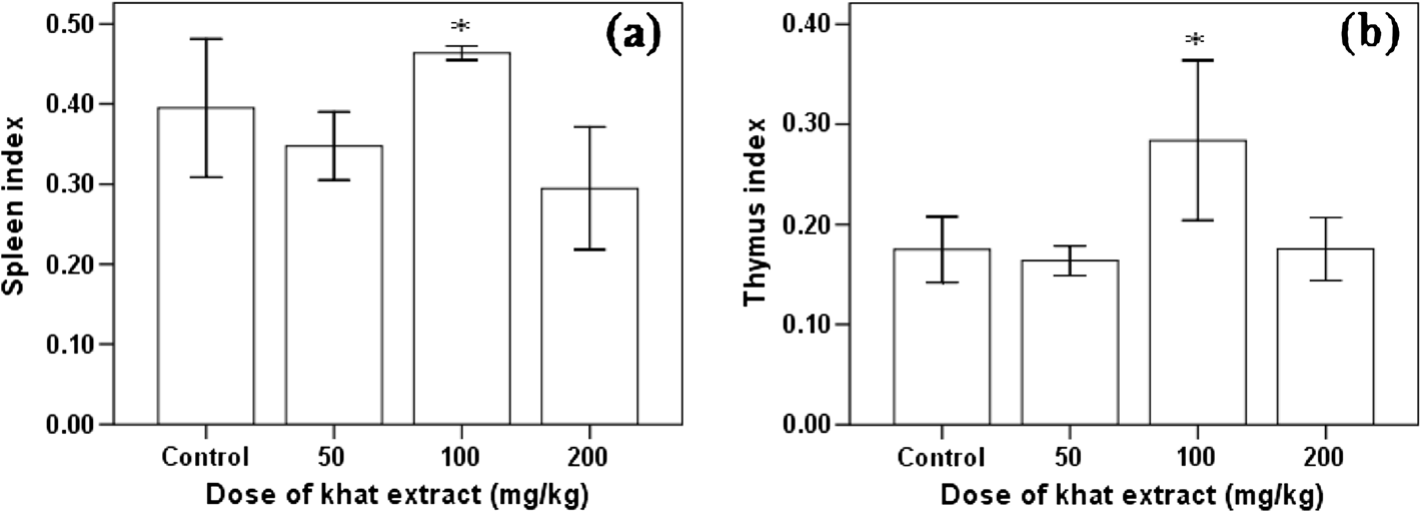
**Spleen (a) and thymus (b) index (Mean ± SEM) of Swiss albino mice (n = 6) treated with crude khat extract daily for 4 weeks.** Values with asterisk are significantly different (ANOVA, Tukey’s HSD *post-hoc* test) from values of control.

Likewise, significant increment (P < 0.01) of spleen and thymus was observed in mice treated with khat extract at dose 100 mg/kg BW (Figure 3). But, liver and kidney weight didn’t show significant difference (P > 0.05) between khat treated and the control mice, though increment of dose was associated to increment in weight of liver and kidney (Table 1).

**Table 1:** **Relative organ weight (Mean ± SEM) of mice (n = 6) treated with different doses of cathinone and crude khat extract daily for 4 weeks**

**Test substances**	**Group**	**Organ weight (mean ± SEM)**	
		**Liver**	**Kidney**
	**Control**	**5.26 ± 0.069**	**1.376 ± 0.09**
Cathinone (mg/kg)	0.625	5.38 ± 0.058	1.35 ± 0.075
	1.25	5.59 ± 0.029	1.38 ± 0.014
	2.5	6.1 ± 0.153	1.44 ± 0.11
	5.0	6.65 ± 0.167	1.9 ± 0.096
Khat (mg/kg)	50	6.02 ± 0.15	1.49 ± 0.04
	100	5.89 ± 0.023	1.38 ± 0.03
	200	6.1 ± 0.18	1.8 ± 0.1

### Effect of khat and cathinone on hematological and biochemical parameters

Mice treated with different dose of khat and cathinone showed change on some hematological parameters. As treatment dose increased, the level of RBC showed increment, but there was no significant difference (P > 0.05) between khat and cathinone treated (under all doses) and the control mice. To the contrary, count of platelets, MCV and MCHC were significantly reduced (P < 0.05) in mice that received higher dose of cathinone (2.5 and 5 mg/kg for MCH and MCHC, and all doses, 0.625 to 5 mg/kg for platelet). Also in mice received 100 and 200 mg/kg of khat extract, platelet count was significantly reduced (P < 0.05), while MCV was affected only under highest dose of khat extract (200 mg/kg) (Table 2).

**Table 2:** **Effect of cathinone and crude khat extract on hematological parameters (mean ± SEM) of mice (n = 6)**

**Dose (mg/kg)**	RBC*106/μL	**HB (g/dL)**	**HCT (%)**	**MCV (FL)**	**MCH (pg)**	**MCHC (g/dL)**	Platelet*10 ^**3**^ **/μL**
Cathinone							
Control	8.07 ± 0.3	12.82 ± 0.47	44.3 ± 0.67	54.85 ± 0.03	15.76 ± 0.047	30.91 ± 0.13	884 ± 26
0.625	7.68 ± 0.15	12.51 ± 0.27	40.6 ± 0.14	52.82 ± 0.46	16.28 ± 0.042	30.82 ± 0.2	694 ± 16*
1.25	8.72 ± 0.15	13.7 ± 0.23	43.5 ± 0.78	52.2 ± 0.00	15.63 ± 0.067	30.1 ± 0.0	628 ± 12*
2.5	8.92 ± 0.64	12.1 ± 1.04	40.7 ± 0.23	51.4 ± 0.11**	15.25 ± 0.086	29.65 ± 0.2*	581 ± 23*
5	8.07 ± 0.31	12.12 ± 0.04	44.2 ± 0.67	51.8 ± 0.03**	15.55 ± 0.047	28.7 ± 0.13*	509 ± 48*
Khat							
50	8.3 ± 0.72	12.7 ± 1.11	43.3 ± 1.06	52.06 ± 1.5	15.67 ± 0.31	29.52 ± 0.53	670 ± 12.7
100	8.7 ± 0.25	13.6 ± 0.45	46.1 ± 1.94	52.9 ± 1.36	15.23 ± 0.02	29.42 ± 0.25	639 ± 27.6*
200	8.63 ± 0.37	12.66 ± 0.25	43 ± 1.71	51.66 ± 1.4*	15.63 ± 0.07	29.53 ± 0.15	543 ± 42*

NB: Values with asterisk are significantly different: * = P < 0.05, ** = P < 0.01 (ANOVA, Tukey’s HSD *post-hoc* test) from values of control.

**Figure 4:**
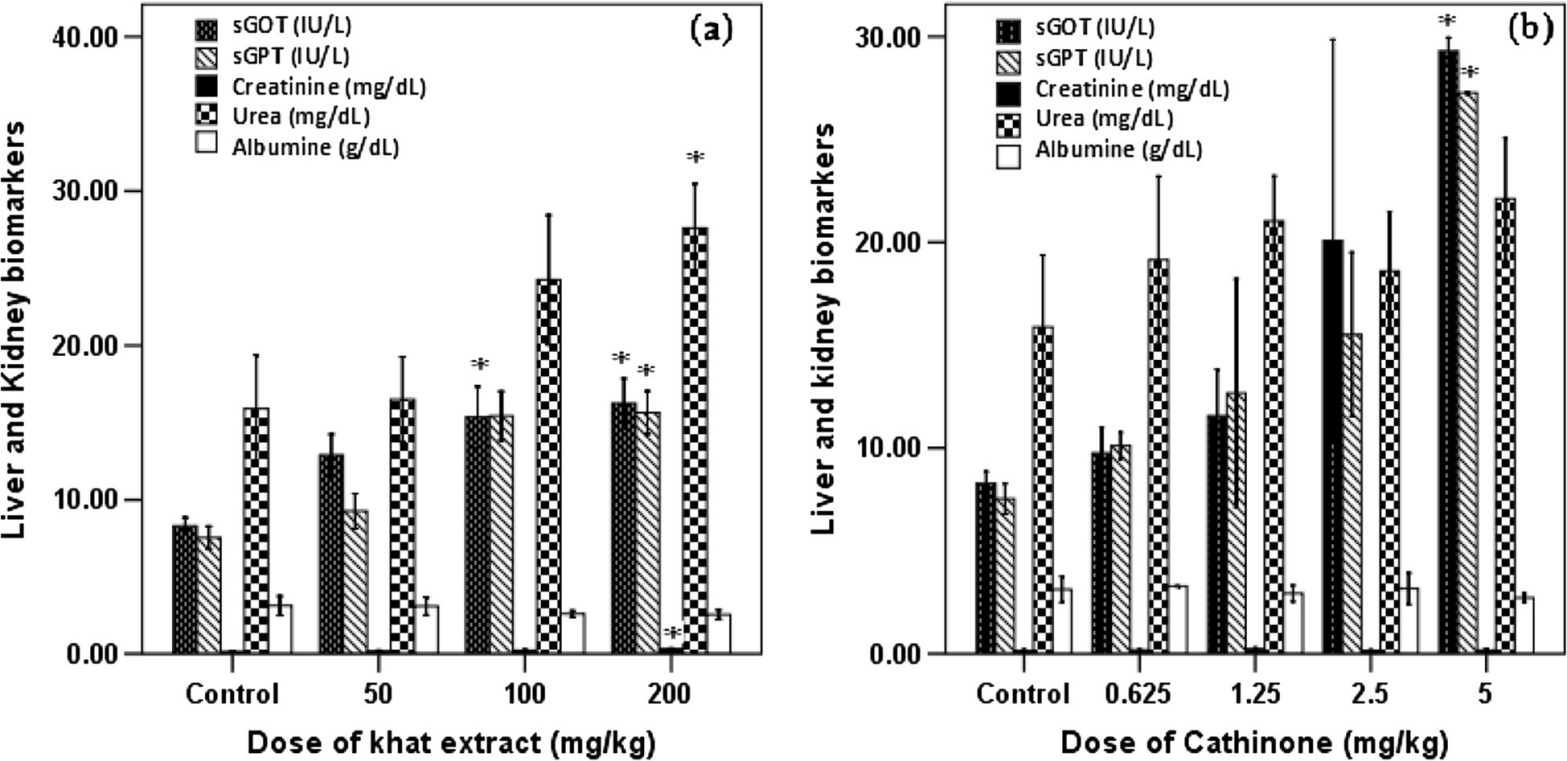
**Liver and kidney biomarkers profile of Swiss albino mice (n = 6) treated with crude khat extract (a) and cathinone (b) daily for 4 weeks.** Values with asterisk are significantly different (ANOVA, Tukey’s HSD *post-hoc* test) from values of control.

The study elaborated the fact that khat and cathinone affect liver and kidney functions. Accordingly, in mice treated with different doses of khat (100 and 200 mg/kg) and cathinone (5 mg/kg) significantly higher (P < 0.05) release of sGOT enzyme was observed. Though higher level of sGPT was measured in mice treated with highest doses of khat and cathinone, significant difference (P < 0.05) was observed only in mice treated with 200 mg/kg of khat extract and 5 mg/kg of cathinone, while albumin level was not significantly different (P > 0.05) between mice treated with the test substances and the control. Likewise, level of kidney biomarkers, creatinine and urea were only affected in mice treated with crude extract of khat. In mice that received higher dose of khat (200 mg/kg), significant increment (P < 0.05) of creatinine and urea was observed as compared to the control group. Cathinone of the chosen dose didn’t show effect on all kidney biomarkers, as there were no significant differences (P > 0.05) on the level of creatinine and urea between the treated and control mice (Figure 4).

#### Effect of khat and cathinone on immunity

**Figure 5:**
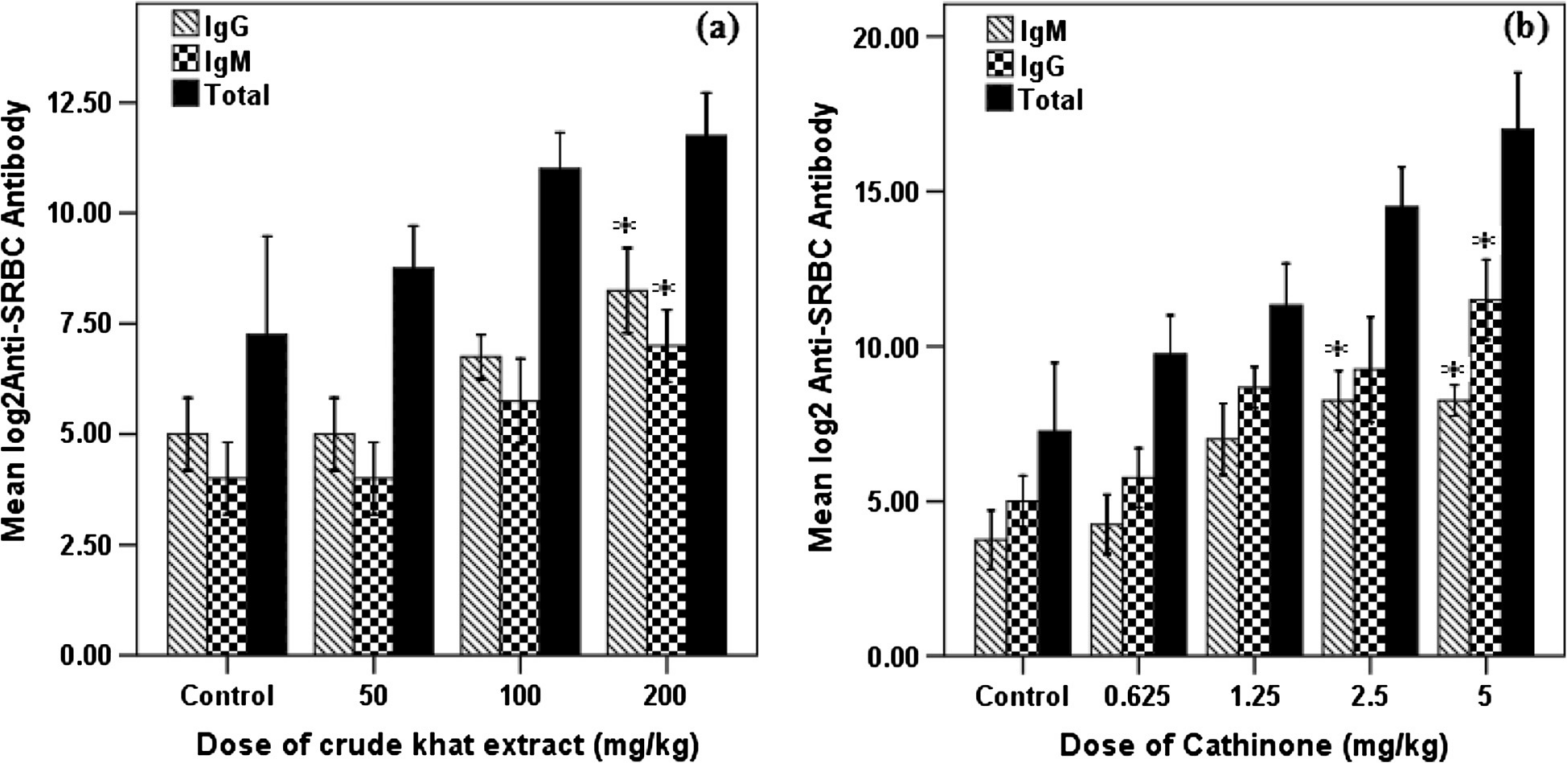
**Anti-SRBCs IgM and IgG titer (mean ± SEM) of Swiss albino mice (n = 6) treated with crude khat extract (a) and cathinone (b) daily for 4 weeks.** Values with asterisk are significantly different (ANOVA, Tukey’s HSD *post-hoc* test) from values of control.

Crude extract of khat and cathinone share some property on antibody production. It was observed that when dose of cathinone increases (at 2.5 and 5 mg/kg), significantly higher (P < 0.001) level of antibody production was observed. Moreover, specific anti-SRBC antibodies: IgG and IgM titer was shown to positively associate to dose of cathinone and khat (Figure 5). As doses of the two test substances increase, rise of the anti-SRBC antibodies titer was observed. But significant increment (P < 0.05) of IgM and IgG level was only observed in mice treated with 200 mg/kg of crude khat extract. Cathinone was associated to significant rise of IgM (P < 0.01) in mice treated with higher doses (2.5 and 5 mg/kg), while significant rise (P < 0.01) of IgG was observed at highest dose (5 mg/kg) (Figure 5).

In mice treated with different doses of khat extract, significantly higher (P < 0.001) level of cellular immune response or DTH (delay type hypersensitivity) was observed at 50 and 100 mg/kg dose, but as dose of the extract increased (200 mg/kg), this arm of the immune response diminished. On the other hand, under all doses of cathinone used, cellular immune response showed significant induction (P < 0.01) (Table 3).

**Table 3:** **Delay type hypersensitivity responses (Mean ± SEM) in mice (n = 6) treated with crude khat extract and cathinone**

**Substance**	**Dose (mg/Kg)**	**DTH response (mm) mean paw edema**
Cathinone	Control	4.75 ± 0.857
	Positive control (Dexamethason)	0.04 ± 0.02
	0.625 mg/kg	4.71 ± 0.57
	1.25 mg/kg	5.1 ± 0.51
	2.5 mg/kg	6.06 ± 0.37*
	5 mg/kg	6.56 ± 0.97**
Khat	50 mg/kg	8.06 ± 0.91**
	100 mg/kg	7.26 ± 1.17**
	200 mg/kg	3.2 ± 0.775*

NB: Values with asterisk are significantly different (ANOVA, Tukey’s HSD *post-hoc* test) from values of control: * = P < 0.01, ** = P < 0.001.

**Figure 6:**
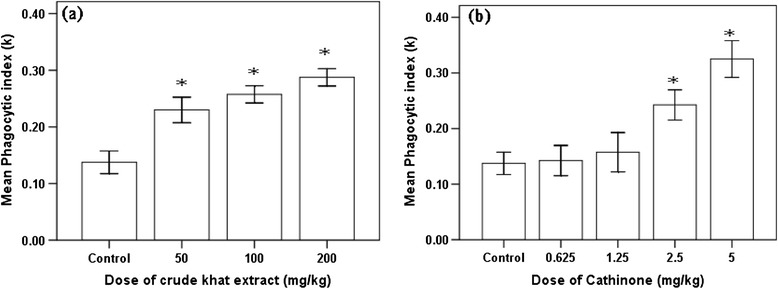
**Phagocytic index (mean ± SEM) of Swiss albino mice (n = 6) treated with crude khat extract (a) and cathinone (b) daily for 4 weeks.** Values with asterisk are significantly different (ANOVA, Tukey’s HSD *post-hoc* test) from values of control.

Effect of the two test substances on the phagocytic activity of reticuloendothelial system was similar to the pattern demonstrated for the humeral immunity. That is, as dose of cathinone increases (2.5 and 5 mg/kg) significantly higher (P < 0.001) phagocytic index than the control mice was observed. However, in all group of mice treated with different doses of crude khat extract (50, 100 and 200 mg/kg) significantly higher (P < 0.001) level of phagocytic index was measured (Figure 6).

**Figure 7:**
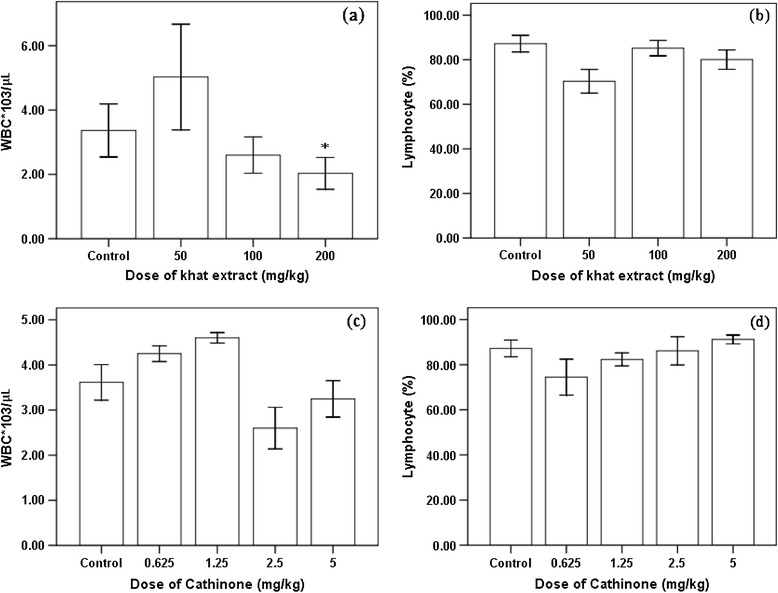
**WBC count (a and c) and lymphocyte level (b and d) of Swiss albino mice (n = 6) treated with khat extract and cathinone daily for 4 weeks.** Value with asterisk is significantly different (ANOVA, Tukey’s HSD *post-hoc* test) from values of control.

In mice chronically treated with khat, significant reduction (P < 0.01) of whole WBC count was observed at higher dose (200 mg/kg). Though there was increased level of WBC in mice that received 50 mg/kg of khat, it was not significantly different (P > 0.05) from the control. The chosen dose of khat and cathinone didn’t affect lymphocyte level in the mice as there was no significant differences (P > 0.05) observed between treated and the control (Figure 7).

**Figure 8:**
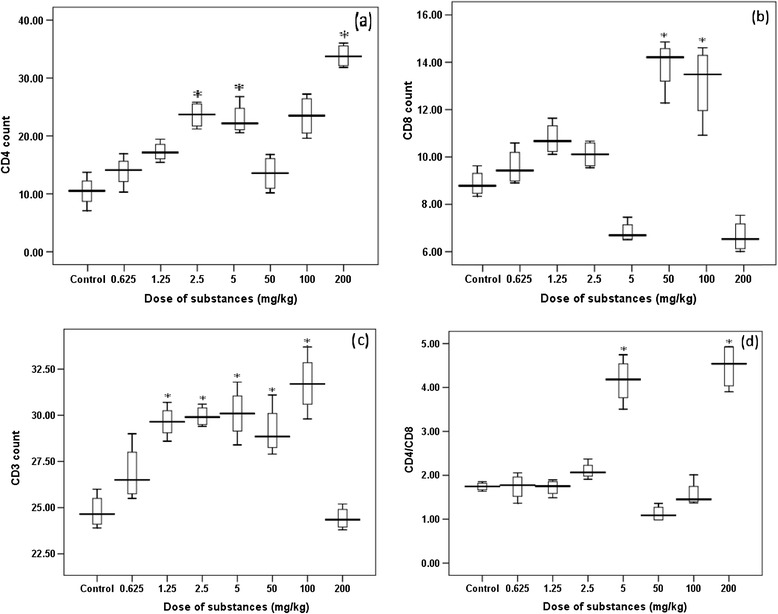
**Box plots showing T lymphocyte populations: CD4** ^**+**^ **count (a), CD8** ^**+**^ **(b), CD3** ^**+**^ **(c) and CD4/CD8 ratio (d) of Swiss albino mice (n = 6) treated with khat extract (50, 100 and 200 mg/kg) and cathinone (0.625, 1.25, 2.5 and 5 mg/kg) daily for 4 weeks.** Value with asterisk is significantly different (ANOVA, Tukey’s HSD *post-hoc* test) from values of control.

CD4^+^ count was significantly increased (P < 0.05) with increase in dose of the crude khat extract (200 mg/kg) and cathinone (2.5 and 5 mg/kg). In mice treated with khat extract of doses 50 and 100 mg/kg, significant rise in CD8^+^ level was observed. The test substances positively modulated increment in CD4^+^ and CD8^+^ cells number, while reduction in CD8^+^ cells count was encountered in mice that has received higher dose of khat (200 mg/kg). Unlike khat, cathinone didn’t strongly affect count of CD8^+^, as there was no significant difference between the treated and the control. CD3^+^ count showed significant increment (P < 0.05) as doses of cathinone and khat increased, but at higher dose (200 mg/kg) of khat there was reduction in count. CD4^+^/CD8^+^ ratio was significantly higher (P < 0.05) in mice treated with highest dose of khat and cathinone (200 and 5 mg/kg, respectively) (Figure 8).

## Discussion

The doses of khat extract (50-200 mg/kg) used in this study was based on toxicity test performed by Al-meshal et al. [14]. Since these doses were reported to induce histopathological effects such as hyperplasia and necrosis of lymphoide organs in mice chronically treated for a minimum of 4 weeks [14], even lesser doses (25-100 mg/kg) can cause chromosomes aberration and disturbed metaphase and anaphase of cell division [21]. Thus, it is believed that the chosen dose of khat extract could show any change including physical, biochemical, hematological and immunological parameters. Accordingly, the methanolic extract of khat (≤200 mg/kg) used in this study didn’t cause body weight reduction, organ (liver and kidney) weight increment, and any sign of toxicity on the khat treated mice, unlike other reports [14,22].

Based on the extraction protocol followed, doses administered and model animal used, different authors reported the direct effect of khat on hematological parameters [23,24]. In our case, using methanol as extraction solvent and dose from 50-200 mg/kg in Swiss albino mice, khat has shown negative effect on total WBC and platelet counts, but not on other hematological parameters. Even though there was no weight difference in liver and kidney of khat and cathinone treated mice and the control, in agreement to other reports [25,26] liver and kidney functions were strongly affected. This implies that, even at relatively lower dose of khat (100-200 mg/kg), but higher dose of cathinone (5 mg/kg) compared to other studies [25,26], there was a rise in the level of liver enzymes and biomarkers of kidney while the level of albumin was found reduced. This might be due to involvement of liver and kidney in metabolism and elimination of khat, respectively.

With the assumption that khat of 50-200 mg/kg dose could affect lymphoid organs [14], the assessment made on weight of spleen and thymus, the immune organs, showed significant increment in weight at relatively lower doses of khat extract and cathinone. These two organs play a major role in immune system. Thymus is a site where T lymphocytes get trained to differentiate self from non-self, mature and then flow into blood circulation [27]. While, spleen is an organ where immune cells, mainly phagocytic cells bring antigen, interact with, and then present to T lymphocytes [27]. Thus, increment in weight of the two immune organs could be an indicator for immune stimulating potential of khat and cathinone.

Also, in mice treated with lower dose of khat and cathinone, no changes were observed in hematological and biochemical parameters. Rather immune responses such as Phagocytic activities of reticulo-endothelial systems, humeral, and cellular immune response to different pattern of challenges in mice were higher at lower dose of the two test substances. This implies that khat has immune potentiating property without causing common harm when it is consumed at lower dose for longer period.

Even though, studies conducted on effect of khat on immune system are very limited, khat chewers were reported to have increased lymphocyte counts and percentage of CD4^+^ cells [28]. In support of the epidemiological data reported by Abuye *et al*. [28], CD4^+^ count and lymphocyte percentage of mice treated with khat and cathinone were found increased with dose dependent manner in the current study, though higher dose of cathinone didn’t cause change. Other *in vitro* study showed that cathinone mediate B-lymphocyte proliferation, cytotoxic T-lymphocyte induction, stimulate lymphocyte proliferation and mediate IL-2 production [29]. In agreement to the reports from the above *in vitro* study, cathinone used in mice experimentation was positively associated to elevated level of antibody titer and DTH in chronically treated and sensitized mice. Likewise, dose dependent increment in antibody titer and cellular response was documented in mice treated with the parent compound (khat). Though there was an increasing level of CD8^+^ count and cellular immunity, as dose of khat and cathinone increases, reduction was observed.

Lymphocytes are cells involved in adaptive immunity and subdivided into B, T, and NK lymphocytes [30]. In mice treated with almost all doses of the test substances (khat and cathinone), level of T helper cells or CD4^+^ was significantly higher than the control. Functionally CD4^+^ cells are vital in assisting other WBCs in immunologic processes, including maturation of B cells into plasma cells and memory B cells, and activation of cytotoxic T cells and macrophages. Increased level of B lymphocytes (IgG and IgM anti-SRBC antibody titer) in all mice received khat and cathinone could be facilitated by direct interaction of B cells with antigen (Ag) or through indirect activation by T-helper cells. Increased level of CD4^+^ in mice from the challenge supports the second notion in which B cells differentiated into plasma cells through facilitation of T cell dependent humeral immune response.

Helper T cells become activated when they encounter Ag coupled to MHC class II molecules, which are expressed on the surface of antigen presenting cells (APCs) such as macrophages, dendritic cells and B cells. Once activated, they divide rapidly and secrete cytokines that regulate immune responses. Helper T cells can differentiate into the two major subtypes of cells Type 1 and Type 2 helper T cells [19,31]. Helper T cells are the host immunity effectors against intracellular pathogens. The main effectors cells of type 1 helper T cells (Th1) immunity are macrophages, CD8^+^ T cells, IgG B cells, and IFN-γ CD4^+^ T cells [32]. IFN-γ secreted by CD4^+^ T cells can activate macrophages to phagocytose and digest intracellular pathogens [32]. Increased phagocytic activities of reticulo-endothelial systems in mice treated with higher dose of khat and cathinone thus suggests further differentiation of CD4^+^ T cells into Th1 helper cells. This was more evidenced by increased incidence of delay type hypersensitivity reaction in almost all mice received khat and cathinone. Since Th1 over activate against auto-antigens that will cause delayed-type hypersensitivity [32]. Thus, from the fact that higher level of antibody titer secretion associated to Th2 T cells, and higher phagocytic activity observed at all selected doses of khat and cathinone, there could be possibility of stimulation of both Th1 and Th2 cytokines in treated mice.

Khat is known to contain compounds such as alkaloids, terpenoids, flavonoids, sterols, glycosides, tannins, amino acids, vitamins and minerals [33]. Some of these compounds, such as alkaloids and flavonoids, are the most common plant metabolites that widely affect immune system in various ways. According to Middleton [34], flavonids are able to activate immune cells such as mast cells, basophils, neutrophils, eosinophils, T and B lymphocytes, macrophages and others. Also several alkaloids have been documented for having immunomodulatory effects in humans [35-37]. Thus, alkaloids in khat that are supposed to play stimulatory role could be partially responsible for immune-modulation.

Recently, researches focusing on assessment of immuno-potentiating substances to enhance the efficacy of pre-existing drugs or identification of immune-suppressant compounds capable of down-regulating excessive undesirable immune response against pathogen to improve hosts condition and ensuring survival are becoming part of the new approaches of combating some diseases [38,39]. With this regard, the use of relatively lower dose of cathinone, 0.625 and 1.25 mg/kg, which have limited negative effect on physical, emotional, physiological, hematological and biochemical parameters could be potential area to be further evaluated for possible use of the substance as an alternative option in such fields.

## Conclusion

Chronic exposure to relatively lower doses (50-100 mg/kg) of khat, with lower effect on physical and physiological conditions of mice, has immune activating potential on phagocytic activity of reticulo-endothelial system, humeral and cellular immune responses. But, as dose increases suppression of cellular immune response occur. Cathinone also share all immune modulating property of its parent compound, khat, but with intense strength.

## Additional file

Additional file 1:
**NMR image of cathinone oxalate; proton and electron assay.**

## Abbreviations

μg/ml: Microgram per ml
Alb: Albumin
ANOVA: Analysis of variance
CBC: Complete blood cells count
CD3^+^: Cluster differentiation 3
CD4^+^: Cluster differentiation 4
CD8^+^: Cluster differentiation 8
Cr: Creatinine
DTH: Delay type hypersensitivity
EPHI: Ethiopia Public Health Institute
FL: Femtoliters
g/dL: Gram per deciliter
Glu: Blood glucose
Hb: Hemoglobin
HCT: Hematocrit
IgG: Immunoglobuline type G
IgM: Immunoglobuline type M
MCH: Mean corpuscular hemoglobin
MCHC: Mean corpuscular hemoglobin concentration
MCV: Mean corpuscular volume
mg/dl: Milligram per deciliter
NMR: Nuclear magnetic resonance
Pg: Petagram
RBC: Red blood cells
Rf: Retardation factor
SEM: Standard error of mean
sGOT: Serum glutamic oxaloacetate transaminase
sGPT: Serum glutamic pyruvic transaminase
WBC: White blood cells
