# Supplementary figures and images for: Evaluation of immunomodulatory activities of methanolic extract of khat (Catha edulis, Forsk) and cathinone in Swiss albino mice
Source: BMC Immunol. 2015 Feb 22;16:9. doi: 10.1186/s12865-015-0072-5 (PMC4341871; doi:10.1186/s12865-015-0072-5)

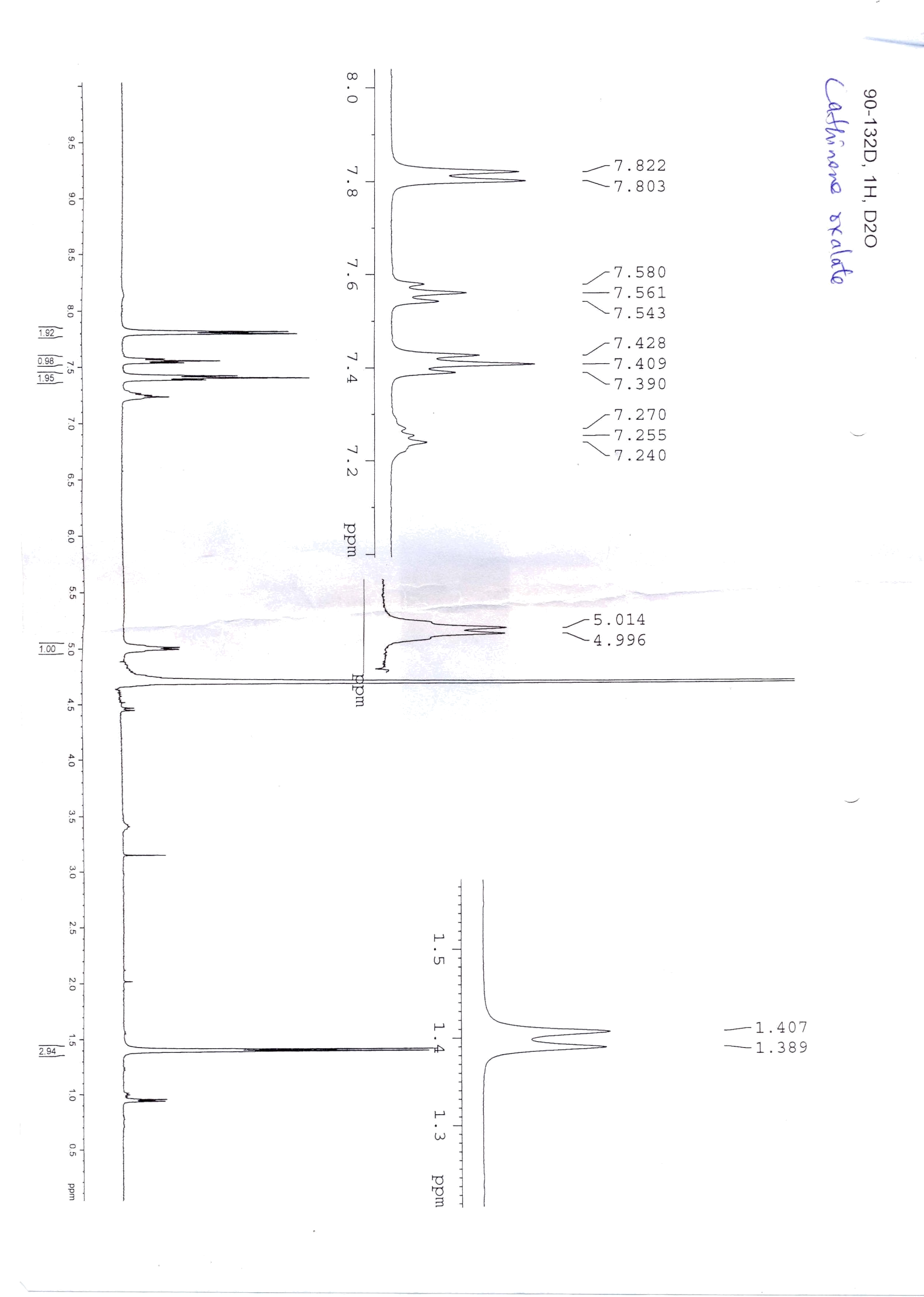


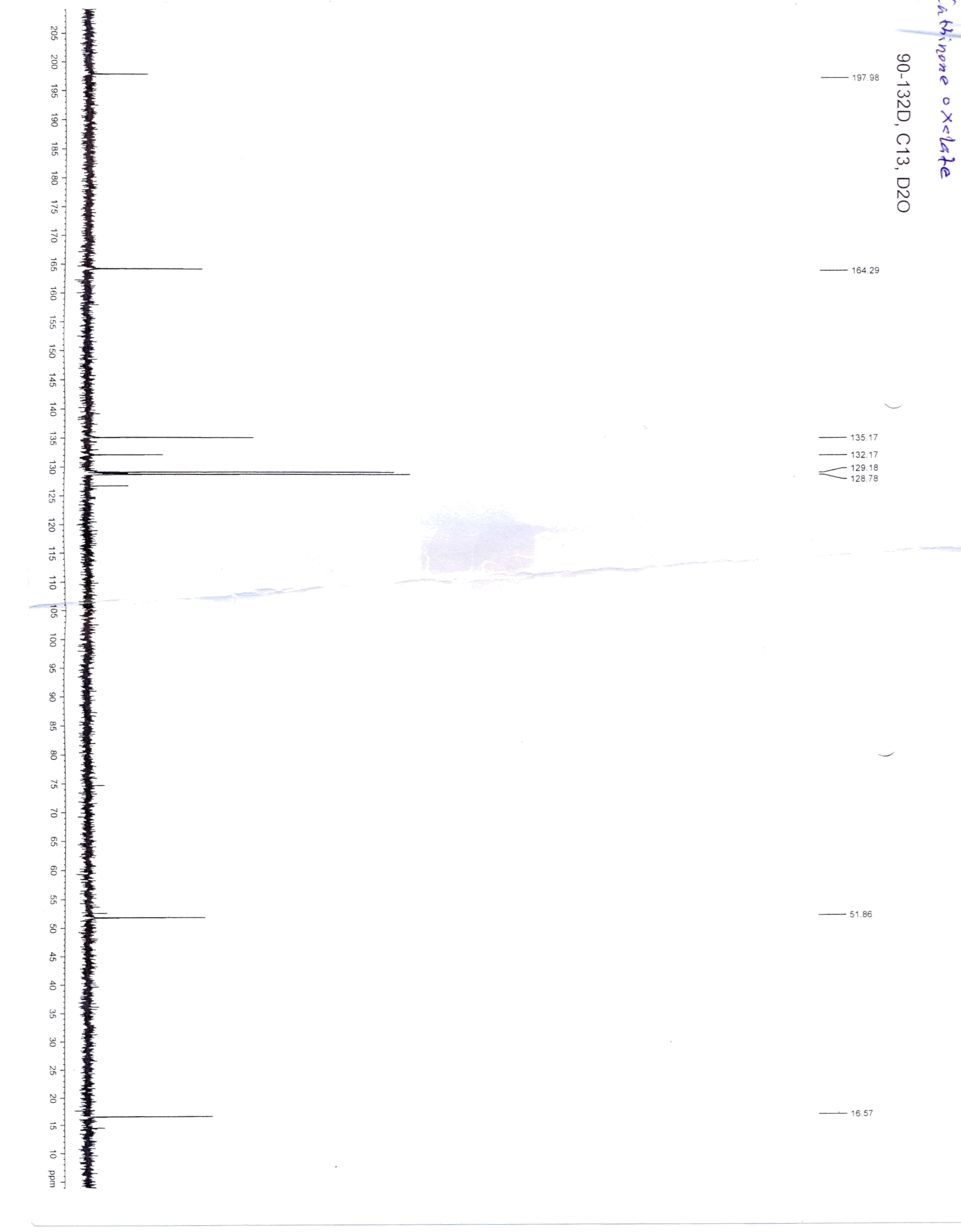


Figure 1 NMR image of cathinone oxalate; proton and electron assay

Supplement: Additional file 1: — NMR image of cathinone oxalate; proton and electron assay. [file 12865_2015_72_MOESM1_ESM.doc]
